# Statin-Associated Muscle Symptoms and Myotoxicity: A Clinically Oriented Narrative Review with a Practical Prevention, Evaluation, and Management Algorithm

**DOI:** 10.3390/medicina62061134

**Published:** 2026-06-10

**Authors:** Francisco Epelde

**Affiliations:** 1 Internal Medicine Department, Parc Taulí Hospital Universitari, Institut d’Investigació i Innovació Parc Taulí (I3PT-CERCA), Universitat Autònoma de Barcelona, 08202 Sabadell, Barcelona, Spain; fepelde@gmail.com; 2 Internal Medicine, Aptima Centre Clinic, 08221 Terrassa, Barcelona, Spain

**Keywords:** statins, statin-associated muscle symptoms, SAMS, myopathy, myositis, rhabdomyolysis, nocebo effect, drucebo, statin intolerance, anti-HMGCR myopathy, creatine kinase

## Abstract

*Background and Objectives*: Muscle symptoms are the most visible adverse event attributed to statins, but terminology is often imprecise. Most patients report myalgia or nonspecific aches, whereas objective myopathy, inflammatory or necrotizing myositis, rhabdomyolysis, and anti-HMGCR immune-mediated necrotizing myopathy are uncommon and clinically distinct entities. To provide a clinically oriented narrative synthesis of statin-associated muscle symptoms (SAMS) and severe statin-associated myotoxicity, and to propose a practical prevention, evaluation, and management algorithm. The classification of muscle events is used to standardize terminology and avoid diagnostic confusion, not to create a new formal taxonomy. *Materials and Methods*: A clinically oriented narrative review was performed using PubMed, Google Scholar, and major society documents published from January 2021 to April 2026. Eligible sources addressed SAMS, statin myopathy/myositis, rhabdomyolysis, anti-HMGCR immune-mediated necrotizing myopathy, nocebo/drucebo effects, pharmacogenetics, drug interactions, diagnosis, or management. The final evidence set comprised 55 verifiable sources, including blinded randomized or n-of-1/crossover evidence; meta-analyses; clinical statements and reviews; pharmacovigilance analyses; pharmacogenetic guidance; mechanism-focused reviews; anti-HMGCR series; and lipid-lowering guideline/treatment studies. Because the review was narrative, no pooled estimate or formal PRISMA screening log was generated. *Results*: Blinded evidence indicates only a small absolute excess of muscle pain with statins, concentrated mainly in the first year of therapy, and that most muscle symptoms reported during statin therapy are not pharmacologically caused by the statin. N-of-1 and crossover trials show that symptom intensity is often similar during statin and placebo periods, consistent with an important nocebo/drucebo contribution. Severe muscle toxicity can nevertheless occur, especially when systemic statin exposure is increased by a high dose, interacting drugs, frailty, renal or hepatic impairment, hypothyroidism, transporter or metabolic genotypes, or intense unaccustomed exercise. Statin choice matters chiefly through dose, pharmacokinetics, and interaction burden. *Conclusions*: SAMS are common as reported clinical problems, but confirmed statin-caused muscle injury is substantially less frequent than routine clinical attribution suggests. Permanent discontinuation should be reserved for carefully assessed cases. A structured approach—baseline risk assessment, selective CK measurement, exclusion of alternative causes, correction of modifiable risks, dechallenge/rechallenge, statin switching, dose reduction, and combination with non-statin therapy—preserves cardiovascular benefit while protecting the rare patient with genuine toxicity.

## 1. Introduction: Scope, Definitions, and Epidemiology

Statins remain foundational drugs for the prevention of atherosclerotic cardiovascular disease. Their clinical value is not in dispute; what is often disputed in daily practice is whether muscle pain in a statin user should be interpreted as statin toxicity. This distinction matters. If every ache that appears after a prescription is considered a pharmacological injury, many patients will stop a treatment that prevents myocardial infarction, stroke, and cardiovascular death. If, conversely, severe weakness, marked creatine kinase (CK) elevation, or autoimmune necrotizing myopathy is ignored, rare but important harms may be missed.

The modern literature supports a balanced position: statin-associated muscle disease exists, but it is much less common than many patients and clinicians believe. Observational practice commonly reports SAMS or statin intolerance in approximately 5–15% of patients, with higher estimates in some settings depending on how symptoms are elicited [1,2,3]. These observational estimates describe symptom burden rather than confirmed causality. In contrast, the Cholesterol Treatment Trialists’ Collaboration individual-participant meta-analysis found only a modest excess of muscle symptoms with statin therapy, largely in the first year, and concluded that more than 90% of muscle symptoms reported by participants allocated to statins were not caused by the statin [4]. A 2026 meta-analysis of double-blind randomized trials designed to evaluate adverse effects attributed in product labels reached a similar conclusion: many events listed or feared in routine care cannot be causally assigned to statins at the perceived rates [5].

This does not mean that patient symptoms are imagined. Pain is real even when it is not pharmacologically caused by the drug. The SAMSON and StatinWISE trials are particularly important because they studied patients who believed they had statin-related symptoms. In both settings, symptom patterns were very similar during statin and placebo periods, demonstrating that expectations and background symptom variability can explain much of the intolerance phenotype [6,7]. This nocebo or drucebo component has been incorporated into recent expert recommendations [8,9].

Precise vocabulary is therefore essential. Statin-associated muscle symptoms (SAMS) is an umbrella term that includes symptoms temporally associated with statin use regardless of causality. Statin myalgia usually describes muscle pain without CK elevation. Myopathy implies objective weakness or biochemical muscle injury. Myositis should be reserved for inflammatory muscle disease or at least objective muscle injury, and rhabdomyolysis for severe myonecrosis with very high CK and risk of renal injury. Anti-HMGCR immune-mediated necrotizing myopathy is a separate autoimmune disease that may persist or progress despite statin withdrawal. Confusing these entities amplifies fear and leads to inappropriate discontinuation (Table 1).

The specific goal of this review is therefore not to introduce a new classification of statin muscle disease, nor to repeat a formal systematic review. Similar recent reviews, clinical perspectives, and society statements have already summarized definitions, prevalence estimates, and management principles [2,3,10,11,12,13,14,15,16,17,18,19]. The added aim here is to integrate those sources with blinded causality evidence, diagnostic uncertainty, CK-based safety thresholds, and pretreatment risk assessment into a single practical algorithm for clinicians. The classification in Table 1 functions as a terminology framework for the algorithm rather than as a proposed new taxonomy.

**Table 1:** Clinical spectrum of muscle events attributed to statins, with practical CK interpretation.

Entity	Typical Presentation	Practical CK Interpretation	Frequency/Message	Practical Response
Myalgia	Aches, cramps, or soreness, often bilateral but nonspecific; no objective weakness.	Normal CK. Minor elevations may reflect exercise or background disease rather than statin injury.	Common in adults; most symptoms in blinded trials are not caused by statins [4,5,6,7].	Assess temporal pattern and other causes; avoid permanent withdrawal after one episode.
SAMS	Symptoms temporally related to statin use, with or without causality.	Normal or mild CK elevation, commonly <4 × ULN; causality is not assumed from symptoms alone.	Useful clinical label, but should not be equated with proven pharmacological toxicity [1,2,3,10].	Use dechallenge/rechallenge, symptom diary, risk-factor correction, and shared decision-making.
Myopathy/myositis	Muscle pain plus objective weakness or biochemical muscle injury.	Usually elevated. CK > 4 × ULN with symptoms suggests clinically relevant injury; some trial definitions use CK ≥ 10 × ULN for myopathy.	Uncommon; risk rises with high exposure, interacting drugs, and vulnerable hosts [15,16,17,18,19,20,21].	Check CK, renal function, thyroid status, and interactions; hold statin if CK is marked or symptoms are severe.
Rhabdomyolysis	Severe muscle pain or weakness, dark urine, systemic illness, or acute kidney injury.	Typically CK > 10 × ULN, often much higher (e.g., >40 × ULN or >10,000 IU/L) with myoglobinuria or renal risk.	Very rare but clinically serious; avoid high-risk drug combinations [22,23,24,25,26,27,28].	Stop statin, hydrate, monitor renal function/electrolytes, and identify precipitants.
Anti-HMGCR IMNM	Progressive proximal weakness, high CK, and persistence or progression after stopping statin.	Marked and persistent CK elevation, often >10 × ULN; does not normalize simply with withdrawal.	Rare autoimmune entity, distinct from ordinary SAMS [29,30,31,32].	Test anti-HMGCR when phenotype fits; refer to neuromuscular/rheumatology; immunosuppression is often needed.

Note: CK thresholds vary across guidelines and laboratories; multiples of the upper limit of normal (ULN) should be interpreted with symptoms, renal function, exercise history, and alternative causes.

## 2. Methodology

This article was designed as a clinically oriented narrative review, not as a formal systematic review or a meta-analysis. Its purpose was to clarify causal attribution and diagnostic uncertainty in statin-associated muscle symptoms, and to develop a practical prevention, evaluation, and management algorithm for everyday clinical use. A formal new classification was not intended; the terminology table is used only to separate common reported symptoms from objectively dangerous entities such as myopathy, rhabdomyolysis, and anti-HMGCR immune-mediated necrotizing myopathy.

The literature search covered publications from January 2021 to April 2026. Searches were performed in PubMed and Google Scholar using combinations of: statin-associated muscle symptoms, SAMS, statin intolerance, statin myopathy, statin myositis, rhabdomyolysis, nocebo, drucebo, n-of-1 trial, simvastatin, atorvastatin, rosuvastatin, pravastatin, pitavastatin, drug–drug interaction, *SLCO1B1*, *ABCG2*, *CYP2C9*, anti-HMGCR, immune-mediated necrotizing myopathy, bempedoic acid, ezetimibe, and PCSK9. Recent guidelines and consensus documents from lipid, cardiovascular, pharmacogenetic, and neuromuscular societies were also reviewed. The date restriction was chosen to maintain a current bibliography, but it was considered a potential source of recency-related selection bias and is explicitly addressed below.

Eligible sources were clinical trials, individual-participant or conventional meta-analyses, n-of-1 or crossover studies, recent clinical statements, pharmacogenetic prescribing guidance, pharmacovigilance analyses, and clinically relevant reviews addressing incidence, causality, mechanisms, diagnosis, risk factors, or management. Publications were excluded from the main evidence synthesis when they were outside the review scope, duplicated information, had insufficient bibliographic detail to support accurate citation, or were case reports used to estimate incidence. Case reports and case series were considered only for rare phenotypes such as anti-HMGCR immune-mediated necrotizing myopathy, where they help illustrate clinical presentation rather than frequency.

To provide transparent numerical information without implying a formal systematic review, the final evidence set was categorized as follows: 55 verifiable sources were included. These comprised 4 blinded randomized, n-of-1/crossover or meta-analytic causality sources [4,5,6,7], 12 statements, reviews, prevalence, or clinical-intolerance sources [1,2,3,8,9,10,11,12,13,14,33,34], 10 pharmacology, statin-specific, exercise, or vitamin-D/risk-modifier sources [15,16,17,18,19,20,21,35,36,37], 6 pharmacovigilance or interaction sources [22,23,24,25,26,27], 4 pharmacogenetic sources [38,39,40,41], 4 mechanism-focused sources [28,42,43,44], 4 anti-HMGCR immune-mediated necrotizing myopathy sources [29,30,31,32], and 11 guideline, lipid-lowering strategy, or discontinuation/outcome sources [45,46,47,48,49,50,51,52,53,54,55].

**Figure 1:**
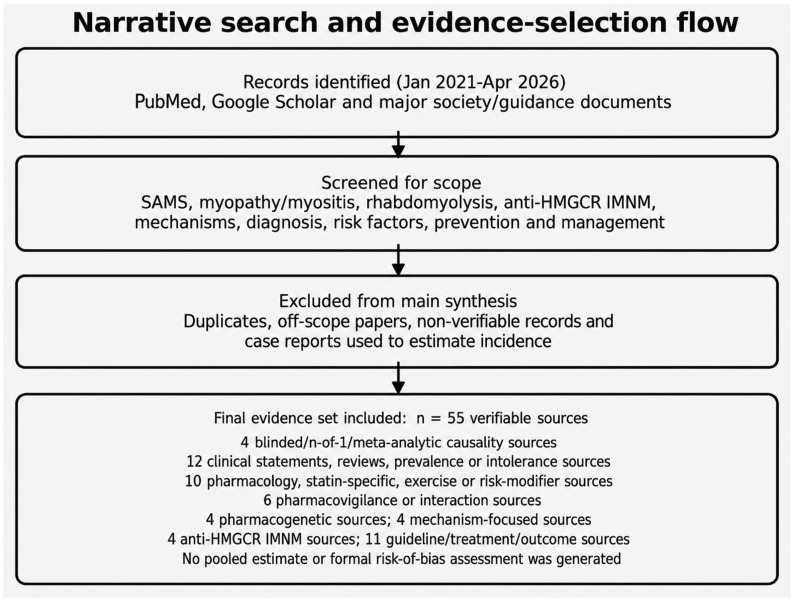
Narrative search and evidence-selection flow. The figure reports the final evidence set (n = 55) by source type and is intended to make the narrative selection process transparent; it is not a PRISMA diagram for a systematic review.

Because this is a narrative review, no reproducible database screening log, formal risk-of-bias assessment, or pooled estimate was generated. The evidence was weighted according to clinical relevance and design hierarchy. Blinded randomized trials and individual-participant meta-analyses were prioritized for causal attribution, whereas pharmacovigilance studies were used for signal detection, and mechanism-oriented reviews were used to summarize biologically plausible pathways. Prioritizing publications from 2021 to 2026 was intended to keep the bibliography current; however, this choice may introduce recency-related selection bias by underrepresenting classic mechanistic, pharmacokinetic, and epidemiological studies. Older foundational work was considered mainly through recent guidelines and reviews rather than being extensively cited. Reference details were checked against bibliographic databases or publisher records before citation. Narrative search and evidence selection flow is presented in Figure 1.

## 3. Frequency and Causal Attribution of Statin-Associated Muscle Disease

Prevalence estimates vary widely because the denominator and definition vary. In observational practice, SAMS or statin intolerance is often reported around 5–15%, and some series report higher values depending on how symptoms are elicited [1,2,3]. However, observational rates combine pharmacological injury, coincidental pain, pre-existing musculoskeletal disease, expectation effects, media exposure, and clinician behavior. The blinded randomized evidence is therefore more informative when the question is causal attribution.

The CTT individual-participant meta-analysis is the key contemporary reference. Across large double-blind trials, statin therapy produced only a small absolute excess of muscle pain or weakness compared with placebo, with most excess occurring during the first year [4]. The practical interpretation is profound: when a patient taking a statin reports muscle symptoms, the symptoms deserve attention, but the prior probability that the statin is the true cause is lower than commonly assumed. The recent Lancet product-label analysis strengthened the same message by showing that adverse effects attributed in labels often overstate what can be proven in blinded randomized comparisons [5].

The statement that most reported muscle symptoms are not pharmacologically caused by statins is therefore a probabilistic interpretation of blinded group-level evidence, not a dismissal of individual symptoms. Population heterogeneity may modify the statin-attributable fraction: trial participants differ from routine clinical patients in age, sex distribution, comorbidity, frailty, background musculoskeletal pain, exercise exposure, polypharmacy, drug-interaction burden, nocebo/drucebo susceptibility, and statin dose or systemic exposure. Therefore, group estimates should not be applied mechanistically to every patient, and individual evaluation remains necessary.

N-of-1 trials provide a complementary patient-level lens. StatinWISE enrolled patients who had previously stopped or considered stopping statins because of muscle symptoms and compared atorvastatin with placebo in repeated blinded periods. Mean muscle symptom scores were not materially worse during statin months than placebo months [6]. SAMSON used periods of statin, placebo, and no tablet; symptom burden was substantially higher with tablets than with no tablet, but similar between statin and placebo tablets [7]. These results do not invalidate symptoms. They show that symptoms alone, without blinded rechallenge or objective injury, can lead to overattribution of causality.

The nocebo/drucebo literature is therefore not a rhetorical device but a clinical tool. Explaining to patients that muscle symptoms are common in adults and that blinded trials show small drug-specific effects can reduce fear, preserve trust, and allow rechallenge [8,9]. This conversation should be handled carefully: telling a patient that pain is not real is wrong; explaining that pain may be real but not caused by the statin is accurate and therapeutic.

Recent clinical reviews and society documents converge on the same approach: evaluate symptoms, look for reversible risk factors, and keep some LDL-lowering therapy whenever possible [2,3,10,11,12,13,14,15,16,17,18,19]. The consequence of overdiagnosis is not benign. Registry and cohort studies in older adults associate statin discontinuation with increased cardiovascular events and mortality, although confounding remains possible [53,54]. The burden of proof should therefore be high before a life-long preventive therapy is abandoned (Table 2).

Observational estimates describe reported symptoms during statin therapy, not confirmed statin-induced muscle toxicity.

## 4. Diagnostic Uncertainty in SAMS

Diagnostic uncertainty is intrinsic to SAMS because there is no single symptom, laboratory test, or imaging finding that proves ordinary statin-attributable muscle pain. CK is useful for detecting objective muscle injury, but most patients with reported SAMS have normal CK. Conversely, mild CK elevations can occur after exercise, viral illness, hypothyroidism, or other muscle diseases and should not automatically be attributed to statins.

Temporal association is necessary but not sufficient. Symptoms that begin soon after statin initiation or dose escalation, improve after withdrawal, and recur with rechallenge are more suggestive than symptoms that are asymmetric, focal, persist unchanged after discontinuation, or have an alternative explanation. Even dechallenge/rechallenge is imperfect when unblinded, because expectations can influence symptom perception. Blinded n-of-1 trials provide stronger causal inference but are rarely feasible in routine practice [6,7,33].

A structured clinical probability assessment is therefore more useful than a binary label. The assessment should document symptom distribution, timing, CK when indicated, previous pain, physical activity, thyroid status, renal and hepatic function, alcohol use, interacting medications, and prior statin exposures. Tools such as symptom diaries and statin-associated muscle symptom clinical indices can standardize the conversation, but they support rather than replace clinical judgment [2,3,8,10,16,17,18,19].

Diagnostic uncertainty should not lead to therapeutic nihilism. Patients with mild aches and normal strength usually merit careful evaluation and rechallenge rather than a permanent intolerance label. Patients with objective weakness, marked or persistent CK elevation, dark urine, systemic illness, or progression after statin withdrawal merit urgent work-up for severe myotoxicity, rhabdomyolysis, or anti-HMGCR immune-mediated necrotizing myopathy [29,30,31,32].

## 5. Statin-Specific Differences in Muscle-Safety Risk

Differences between statins are clinically important, but they should be framed correctly. For ordinary muscle pain with normal CK, the evidence that a single statin is intrinsically much more symptomatic than all others is weaker than many clinicians assume. Differences become more credible when they are explained by dose, systemic exposure, metabolism, transporter dependence, renal clearance, and interacting drugs. In other words, statin selection matters most when it reduces excessive exposure in a vulnerable patient.

Simvastatin and lovastatin are the most interaction-sensitive commonly used statins because they are extensively metabolized by CYP3A4. Strong CYP3A4 inhibitors—for example, clarithromycin, itraconazole, ketoconazole, some HIV or hepatitis C antivirals, and certain calcium channel blockers, depending on dose and statin—can markedly increase statin concentrations. Atorvastatin also depends on CYP3A4, although its dose range and pharmacokinetic profile differ. Pravastatin, fluvastatin, and pitavastatin are less CYP3A4-dependent; rosuvastatin is also less CYP3A4-dependent but is influenced by transporters and renal function. These pharmacological distinctions are why a patient who cannot tolerate one statin often tolerates another [19,20,21,27,38,39,40].

Hydrophilic statins such as pravastatin and rosuvastatin are often assumed to cause fewer muscle symptoms than lipophilic statins such as simvastatin, lovastatin, and atorvastatin. The hypothesis is biologically plausible because tissue distribution differs, but clinical evidence is not uniform. A large observational cohort comparing new users did not find a systematically lower risk of muscular events with hydrophilic versus lipophilic statins at comparable lipid-lowering doses [37]. Thus, switching from a lipophilic to a hydrophilic statin is reasonable in an individual patient, but it should not be presented as a guarantee.

Pharmacovigilance data are useful for signal detection, especially for rare outcomes such as rhabdomyolysis, but they are not incidence studies. Recent analyses of VigiBase and FAERS suggest that reports of rhabdomyolysis vary across statins and are prominent with simvastatin or atorvastatin in many settings [22,23,24]. The interpretation must consider prescribing volume, dose, reporting bias, and comorbidities. Still, the same practical message emerges: avoid high-dose statins in high-risk combinations, be cautious with simvastatin/lovastatin when CYP3A4 inhibitors are used, and consider statins with a lower interaction burden when polypharmacy is unavoidable.

The LODESTAR secondary analysis comparing rosuvastatin with atorvastatin in coronary artery disease did not support a simple narrative that one high-intensity statin is universally safer; safety profiles differed by outcome and patient context [50]. For patients with muscle symptoms, the more useful approach is individualized: reduce intensity, switch molecule, use alternate dosing when needed, and add ezetimibe or other nonstatins to maintain LDL-cholesterol reduction [45,46,47,48,49,50,51,52] (Table 3).

## 6. Mechanisms of Statin-Associated Muscle Symptoms and Muscle Injury

Statins inhibit HMG–CoA reductase and reduce hepatic cholesterol synthesis; however, the same mevalonate pathway also generates nonsterol intermediates that support ubiquinone synthesis, protein prenylation, membrane trafficking, and mitochondrial function. The mechanistic model used in this review is therefore not a single-pathway model. It separates (i) background muscle symptoms and nocebo/drucebo effects, (ii) exposure-dependent toxic stress in susceptible muscle, and (iii) rare autoimmune anti-HMGCR disease [28,42,43,44].

Mevalonate pathway depletion and impaired prenylation. Reduced levels of farnesyl pyrophosphate and geranylgeranyl pyrophosphate may impair prenylation of small GTP-binding proteins involved in cytoskeletal organization, vesicular trafficking, membrane repair, and cell survival signaling. In susceptible patients, this may reduce the ability of muscle fibers to respond to mechanical or metabolic stress. This mechanism is plausible, but it does not, by itself, prove that ordinary myalgia with normal CK represents structural muscle injury.

Mitochondrial bioenergetics, ATP reserve, and morphology. Skeletal muscle depends on oxidative phosphorylation during sustained activity. By reducing ubiquinone/coenzyme Q10 synthesis and altering electron transport, statins may reduce mitochondrial ATP reserves. ATP shortage can impair actin–myosin cycling, sarcolemmal ion pumps, and calcium reuptake by the sarcoplasmic reticulum, thereby promoting fatigue, cramps, or weakness when physiological reserve is low. Experimental studies also describe changes in mitochondrial morphology, including swelling, altered cristae organization, and impaired fusion–fission balance. These changes could amplify vulnerability during high doses, drug interactions, renal or hepatic impairment, hypothyroidism, or intense unaccustomed exercise, but clinical correlation is variable, and supplementation with coenzyme Q10 has not become a universal prevention strategy.

Oxidative stress, calcium handling, and membrane excitability. Impaired electron transport and mitochondrial dysfunction may increase reactive oxygen species. Oxidative stress can damage lipids, proteins, and mitochondrial DNA, activate inflammatory or apoptotic signaling, and reduce membrane stability. Disturbed calcium handling may further increase mitochondrial permeability, protease activation, and contractile dysfunction. Together, ATP depletion, oxidative stress, and calcium overload provide a coherent explanation for true exposure-related myotoxicity, especially when CK elevation or objective weakness is present.

Exposure-dependent toxicity and pharmacogenetics. Clinically, increased systemic statin exposure remains the most actionable mechanism. High dose, rapid dose escalation, CYP3A4 inhibition, OATP1B1 inhibition, renal or hepatic impairment, frailty, hypothyroidism, and variants in *SLCO1B1*, *ABCG2*, or *CYP2C9* can shift a patient from routine exposure toward toxic exposure [15,16,17,18,19,20,21,27,38,39,40,41]. This exposure model explains why myotoxicity is more plausible with interacting drugs or in vulnerable hosts, and why dose reduction, temporary interruption during short interacting courses, switching to a lower-interaction statin, and combining a low-dose statin with nonstatin therapy often resolve symptoms.

**Figure 2:**
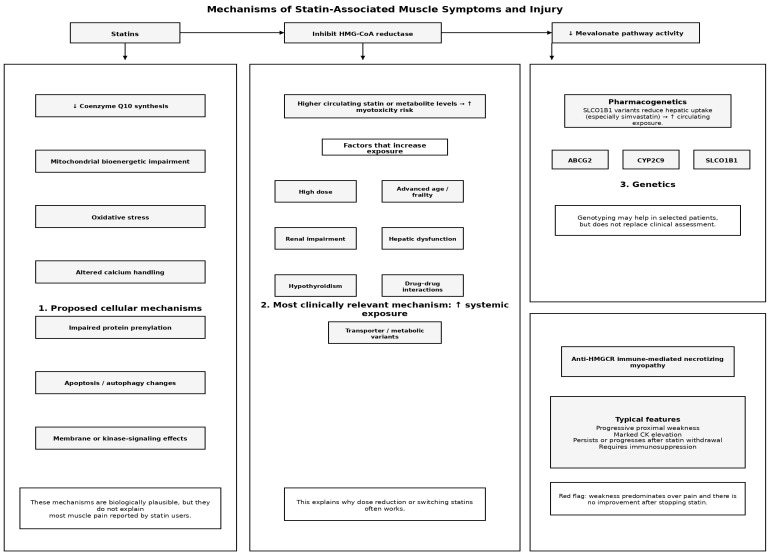
Proposed mechanisms of statin-associated muscle symptoms and injury, emphasizing the distinction be-tween biologically plausible cellular pathways, clinically actionable exposure-related toxicity, and rare autoimmune disease. Arrows indicate direction of effect: ↓ denotes decreased or reduced activity/levels, and ↑ denotes in-creased or elevated activity/levels or risk.

The autoimmune mechanism is qualitatively different. Anti-HMGCR immune-mediated necrotizing myopathy is not simply dose-dependent toxicity. It involves autoantibodies to HMG–CoA reductase, progressive proximal weakness, marked and persistent CK elevation, and progression or persistence after statin withdrawal. Therefore, management requires anti-HMGCR testing, specialist referral and often immunosuppression rather than routine rechallenge [29,30,31,32]. The red flag is not ordinary aching with normal CK; it is progressive weakness, sustained marked CK elevation and failure to improve after stopping the drug (Figure 2).

## 7. Risk Profiles for Reported Symptoms and Objective Muscle Injury

Risk profiling must distinguish between two endpoints: reported muscle pain and severe muscle injury. The patient most likely to report symptoms is not necessarily the patient most likely to develop rhabdomyolysis. Women, older adults, patients with anxiety about medication, those with chronic pain syndromes, and those previously warned about statin side effects may report symptoms more frequently. This does not imply fabrication; it reflects pain biology, background musculoskeletal disease, and expectation effects [1,2,3,8,10,15,34].

The patient most likely to develop objective injury is usually characterized by higher statin exposure, an underlying predisposition, or lower physiological reserve. Recurrent risk factors in recent clinical guidance include advanced age, small body frame or low body weight, frailty, renal or hepatic impairment, untreated hypothyroidism, personal or family history of muscle disease, previous CK elevation, heavy alcohol use, acute illness, perioperative stress, and high-intensity or unaccustomed strenuous exercise [15,16,17,18,19,20,21]. Women and individuals with low body weight appear in many risk lists, but clinicians should avoid reducing the issue to sex alone; polypharmacy, renal function, and dose may be more actionable.

Concomitant drugs are among the most preventable risks. Strong CYP3A4 inhibitors increase exposure to simvastatin, lovastatin, and atorvastatin. Gemfibrozil increases myopathy risk and should generally be avoided with statins when alternatives exist. Cyclosporine, some antivirals, azole antifungals, macrolides, amiodarone, verapamil, diltiazem, colchicine, and antiplatelet combinations in complex patients have all been implicated in interaction effects or pharmacovigilance signals [22,23,24,25,26,27]. The key clinical move is to review the medication list before blaming the statin molecule itself.

Vitamin D deficiency is frequently suspected, but randomized evidence does not support routine vitamin D supplementation as a reliable strategy to prevent SAMS. In a VITAL ancillary study among new statin users, vitamin D supplementation did not prevent statin-associated muscle symptoms or statin discontinuation [35]. Testing and treating vitamin D deficiency may still be appropriate for bone health or symptomatic deficiency, but it should not be oversold as a solution to statin intolerance.

Exercise deserves nuance. Severe unaccustomed exertion can raise CK and may interact with statin exposure in selected cases, yet moderate exercise should not be discouraged. A 2023 JACC study found that prolonged moderate-intensity exercise did not increase muscle injury markers in symptomatic or asymptomatic statin users [36]. Advising patients to stop exercising because they are taking a statin may increase cardiovascular risk and worsen quality of life (Table 4).

## 8. Prevention of Unnecessary Statin Withdrawal

The first prevention strategy is pretreatment assessment. The algorithm was therefore moved earlier, before statin initiation, because recent clinical statements and reviews consistently recommend identifying baseline muscle symptoms, previous intolerance, myopathy history, hypothyroidism, renal or hepatic dysfunction, frailty, heavy alcohol use, exercise patterns, and interacting drugs before attributing later symptoms to statins [2,3,10,15,16,17,18,19]. This change is supported by clinical risk-factor evidence and guidance; however, the manuscript does not advocate universal baseline CK screening. Instead, baseline CK is presented as reasonable only in selected high-risk patients, because routine CK testing in every asymptomatic patient may create unnecessary barriers to proven lipid-lowering therapy.

The second prevention strategy is linguistic. A patient should not leave the consultation believing that muscle pain equals muscle destruction. A practical explanation is: “Muscle aches are common. Statins can cause muscle symptoms in some people, but blinded trials show that most symptoms occurring on statins are not caused by the statin. We will check for danger signs and then find a regimen you can tolerate.” This framing validates symptoms while preserving the possibility of rechallenge.

When symptoms occur, CK is indicated if there is weakness, dark urine, systemic illness, severe pain, symptoms soon after high-dose initiation or dose escalation, renal impairment, interacting drugs, or a phenotype suggesting severe myotoxicity. CK should be interpreted in the clinical context: a normal CK strongly argues against myositis or rhabdomyolysis, whereas marked CK elevation, especially above 10 times the upper limit of normal or persistent after statin withdrawal, requires more urgent evaluation.

The first failed statin should not be the final word. Most patients labeled intolerant can take some statin exposure if rechallenged carefully. Options include briefly stopping until symptoms settle, restarting at a lower dose, changing to a different statin, using a lower-interaction molecule, dosing intermittently in selected patients, or combining a low-dose statin with ezetimibe [2,3,10,16,17,18,19]. The aim is not necessarily the maximum statin dose; it is durable LDL-cholesterol lowering with acceptable tolerability.

Combination therapy is central. RACING showed that moderate-intensity statin plus ezetimibe was noninferior to high-intensity statin monotherapy in patients with ASCVD and produced fewer intolerance-related discontinuations or dose reductions [48]. LODESTAR showed that a treat-to-target strategy can be a reasonable alternative to fixed high-intensity statin therapy in coronary disease [49]. These trials support a practical message: when muscle symptoms threaten adherence, adding nonstatin therapy to a tolerated statin dose may be better than forcing high-intensity monotherapy.

If true statin intolerance persists after trials of at least two statins, nonstatin agents should be used rather than abandoning lipid-lowering. Ezetimibe is the simplest first add-on. Bempedoic acid has outcome data in statin-intolerant patients from CLEAR Outcomes, including a primary prevention analysis [51,52]. PCSK9 monoclonal antibodies and inclisiran achieve substantial reductions in LDL-cholesterol, although access and cost vary. Recent ACC and ESC guidance supports individualized nonstatin therapy when LDL goals are not achieved with tolerated statin therapy [45,46,47].

Finally, severe toxicity should be treated decisively. Statins should be stopped when CK is markedly elevated, when rhabdomyolysis is suspected, or when there is progressive proximal weakness suggesting immune-mediated necrotizing myopathy. Anti-HMGCR testing and specialist referral are appropriate when CK remains high or weakness progresses after withdrawal [29,30,31,32]. The argument that statin-caused muscle toxicity is less frequent than commonly perceived should never become an argument for ignoring objective muscle disease.

## 9. Proposed Prevention, Evaluation, and Management Algorithm

**Figure 3:**
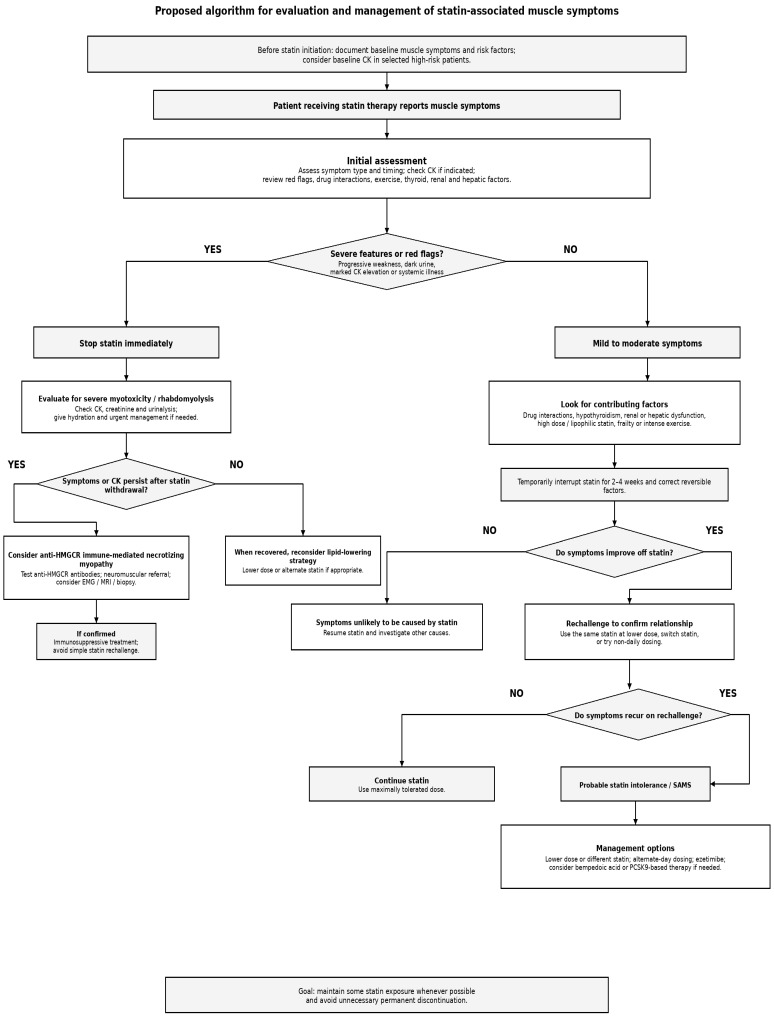
Proposed algorithm for prevention, evaluation, and management of statin-associated muscle symptoms. The algorithm now begins before statin initiation, with baseline symptom/risk assessment and selective CK testing in high-risk patients. This supports prevention and later causal interpretation without recommending routine CK screening for every asymptomatic patient.

1. Assess before statin initiation. Document baseline muscle symptoms, prior statin intolerance, personal or family history of myopathy, thyroid disease, renal or hepatic dysfunction, frailty or low body weight, alcohol use, exercise patterns, and interacting drugs. This pretreatment step is included to reduce later misclassification. Consider baseline CK in selected high-risk patients, but do not use routine CK screening as a barrier to appropriate statin therapy.
2. Confirm the phenotype. Is the complaint pain, cramping, fatigue, weakness, dark urine, or exercise intolerance? Objective weakness and dark urine are red flags; diffuse aches with normal strength are usually lower risk.
3. Measure CK selectively. Check CK when symptoms are severe, objective weakness is present, dark urine or systemic illness occurs, symptoms start soon after high-dose initiation or dose escalation, renal impairment or interacting drugs are present, or severe myotoxicity is suspected. Normal CK supports conservative management; CK above 4 × ULN with symptoms suggests clinically relevant injury, and CK above 10 × ULN or persistent elevation requires urgent evaluation.
4. Search for nonstatin causes. Osteoarthritis, polymyalgia rheumatica, viral illness, hypothyroidism, vitamin D deficiency, strenuous exercise, neuropathy, and chronic pain syndromes are common. Temporal association alone is insufficient.
5. Review the medication list. Look for CYP3A4 inhibitors, gemfibrozil, cyclosporine, colchicine, antivirals, azoles, macrolides, amiodarone, verapamil, or diltiazem. If the interaction is temporary, holding the statin during a short antibiotic or antifungal course may be safer than permanently labeling the patient intolerant.
6. Rechallenge intelligently. After symptom resolution, restart with a different statin, lower dose, or alternate schedule. Consider pravastatin, rosuvastatin, fluvastatin, or pitavastatin when CYP3A4 interactions are central; consider lower-dose atorvastatin or rosuvastatin plus ezetimibe when potency is required.
7. Escalate nonstatin therapy if needed. Persistent inability to tolerate adequate statin exposure should trigger ezetimibe, bempedoic acid, or PCSK9-directed therapy according to cardiovascular risk, LDL-cholesterol level, availability, and guideline recommendations [45,46,47,48,49,50,51,52] (Figure 3).

## 10. Implications for Clinical Communication and Manuscript Framing

The most important editorial point is that statin-associated muscle disease should not be presented as a binary controversy. A binary frame—either statins cause all muscle pain, or they cause none—is clinically harmful. The better frame is probabilistic. Muscle symptoms in statin users are frequent because muscle symptoms are frequent in adults; the statin-attributable fraction is small in blinded data; and the fraction with dangerous muscle injury is much smaller still [4,5,6,7]. This distinction allows clinicians to take symptoms seriously without converting every symptom into a contraindication.

For an article aimed at clinicians, it is useful to separate three decisions. The first is safety: does the patient have severe pain, weakness, dark urine, renal dysfunction, marked CK elevation, or persistence after withdrawal? If yes, the statin should be stopped, and the patient investigated. The second is causality: do symptoms recur reproducibly with different statin exposures and disappear with therapy, after alternative causes have been addressed? If no, permanent intolerance should not be diagnosed. The third is cardiovascular protection: what LDL-lowering regimen can be maintained long term? This may be a full-intensity statin, a lower dose combined with ezetimibe, or a nonstatin regimen when true intolerance is confirmed [2,3,10,45,46,47,48,49,50,51,52].

Patient communication should also avoid inadvertently creating intolerance. Listing muscle pain as an expected event without context can magnify symptom surveillance. A better consent script is balanced: serious muscle injury is rare; aches are common in everyday life; if symptoms occur, the patient should contact the clinician rather than stop therapy permanently; and most patients who develop symptoms can still tolerate a modified regimen. This approach is consistent with the nocebo/drucebo literature and with the growing interest in symptom diaries, n-of-1 approaches, and mobile health monitoring to distinguish background symptoms from drug-attributable effects [6,7,8,9,33].

Finally, the title and discussion should use the term "myositis" carefully. In many clinical conversations, the term is used loosely for any muscle pain on statins, but in a scientific article, it should denote inflammatory or necrotizing muscle disease, or at a minimum, objective muscle injury. Using the term too broadly inflates the perceived danger of statins. A rigorous article can still be persuasive for cardiovascular prevention while being honest about rare harms: ordinary myalgia is usually not statin-caused, pharmacological SAMS is uncommon, severe myotoxicity is very rare, and anti-HMGCR immune-mediated necrotizing myopathy is a distinct, rare autoimmune syndrome requiring specialist care [28,29,30,31,32,42,43,44].

## 11. Limitations

This review has several limitations. First, it is narrative and clinically oriented; it did not use a registered protocol, independent dual screening, formal risk-of-bias assessment, or pooled quantitative estimate. Second, the search prioritized publications from January 2021 to April 2026 and major guidance documents, which improve recency but may introduce selection bias and underrepresent classic mechanistic, pharmacokinetic, and epidemiological studies. Third, Google Scholar searching is less reproducible than database-only systematic searching. Fourth, evidence for mechanisms is heterogeneous and includes cellular, animal, pharmacovigilance, and clinical sources, so mechanistic plausibility should not be equated with causality for most muscle pain. Fifth, trial populations, pharmacovigilance databases, and routine clinical patients differ in age, comorbidity, statin dose, polypharmacy, baseline pain, and expectation effects; therefore, group-level estimates should not replace individualized assessment. Finally, the algorithm is intended as a practical clinical framework and has not been prospectively validated as a decision rule.

## 12. Conclusions

The clinical challenge of SAMS and statin-associated myotoxicity is not whether statins can injure muscle; they can. The challenge is proportionality and diagnostic precision. Ordinary muscle pain in a statin user is common, but blinded evidence shows that most such symptoms are not caused by the statin. True myopathy, inflammatory or necrotizing myositis, rhabdomyolysis, and anti-HMGCR immune-mediated necrotizing myopathy are much less frequent than the public narrative suggests.

The revised message for clinicians and patients should be reassuring but not dismissive. Reassuring, because the absolute risk of serious muscle injury is very low, and most patients can tolerate some statin regimen. Not dismissive, because high-risk patients, interacting drugs, and objective weakness or CK elevation require careful evaluation. The final goal of this review is practical: to provide a terminology framework and a prevention-to-management algorithm that helps clinicians preserve LDL-cholesterol lowering while identifying the rare patient in whom statin toxicity is genuine and clinically significant.

## Figures and Tables

**Table 2:** Frequency and causal attribution of statin-associated muscle disease by evidence type.

Evidence Source	Main Causal Inference	Clinical Implication
Observational practice and routine clinical series [1,2,3,34]	SAMS or statin intolerance is often reported in approximately 5–15% of patients, but these figures combine true pharmacological injury with coincidental pain, background disease, and expectation effects.	Useful for describing reported symptom burden, but they likely overestimate true statin-caused muscle disease.
Large double-blind randomized trials: CTT individual-participant meta-analysis [4]	Statins produced only a small absolute excess of muscle pain or weakness versus placebo, mainly during the first year; most symptoms reported by statin-allocated participants were not statin-caused.	Assess symptoms carefully, but avoid assuming causality from temporal association alone.
N-of-1 and crossover trials: StatinWISE and SAMSON [6,7]	Symptom scores were similar during statin and placebo periods in patients who believed they were statin-intolerant.	Use symptom diaries, dechallenge/rechallenge, and shared interpretation to prevent unnecessary permanent discontinuation.
Recent statements and clinical reviews [2,3,10,11,12,13,14,15,16,17,18,19]	Consensus guidance integrates randomized evidence, pharmacology, and individual risk factors.	Evaluate, correct contributors, rechallenge, and preserve LDL-lowering therapy whenever possible.
Severe myopathy, rhabdomyolysis, and immune-mediated necrotizing myopathy [22,23,24,25,26,27,29,30,31,32]	Objective severe myotoxicity is rare, but red flags include progressive proximal weakness, dark urine, systemic illness, and marked or persistent CK elevation after statin withdrawal.	Stop statin and evaluate urgently; confirmed autoimmune disease requires specialist management and often immunosuppression.
Registry and cohort studies of statin discontinuation [53,54]	Statin discontinuation is associated with higher cardiovascular events and mortality, although residual confounding is possible.	The threshold for permanent statin discontinuation should be high.
Product-label adverse-effect reporting [5]	Muscle adverse effects listed on labels may be broader than what can be proven in blinded randomized comparisons.	Interpret label information in light of randomized evidence and individual patient risk.
Nocebo/drucebo literature [8,9]	Expectations and tablet-taking can contribute substantially to symptom reporting; real pain is not always pharmacologically caused by the statin.	Careful explanation can reduce fear, preserve trust, and facilitate rechallenge.

Abbreviations: CK, creatine kinase; CTT, Cholesterol Treatment Trialists’ Collaboration; SAMS, statin-associated muscle symptoms.

**Table 3:** Practical differences between commonly used statins relevant to muscle symptoms and myotoxicity.

Statin/Group	Pharmacokinetic Features	Muscle Safety Implications	Practical Use When SAMS Is Suspected
Simvastatin/lovastatin	Lipophilic; CYP3A4 substrates; high interaction potential.	Higher concern with strong CYP3A4 inhibitors, cyclosporine, gemfibrozil, and high doses [22,23,24,25,26,27].	Avoid high-dose use; switch if interacting drugs are required.
Atorvastatin	Lipophilic; CYP3A4 substrate; long clinical experience.	Muscle symptoms may relate to dose and interactions; common in reports, partly because of high use [23,24,25].	Lower dose or switch to rosuvastatin/pravastatin/pitavastatin if interaction driven.
Rosuvastatin	Hydrophilic; less CYP3A4 dependence; transporter and renal considerations.	Often useful after symptoms of CYP3A4 statins, but not free of adverse-event reports [24,50].	Use a lower dose in cases of renal impairment or relevant transporter-risk settings.
Pravastatin	Hydrophilic; minimal CYP metabolism.	Lower CYP-mediated interaction burden; less potent LDL lowering per mg.	Useful for rechallenge in polypharmacy or frail patients.
Fluvastatin	Mainly *CYP2C9*; lower-intensity options.	May be tolerated when symptoms occur with stronger statins; limited by potency.	Consider when any statin exposure is better than none.
Pitavastatin	Limited CYP metabolism; potent at low milligram doses.	Potential option in patients with interaction concerns; data are less extensive than for atorvastatin/rosuvastatin.	Consider low-dose rechallenge, often with ezetimibe if LDL target requires.

**Table 4:** Patient and situational factors that increase reported SAMS or objective muscle injury.

Risk Factor or Situation	Likely Mechanism	Prevention or Mitigation
Pre-existing muscle symptoms, personal/family myopathy history, or previous CK elevation	Baseline disorder may be misclassified as statin toxicity, or a latent disorder may be unmasked during therapy.	Document symptoms before treatment; consider baseline CK and specialist input in selected high-risk patients.
Female sex, older age, small body frame/low body weight, frailty	Higher symptom reporting and/or lower physiological reserve; sometimes higher exposure per dose.	Start with appropriate intensity, ask about baseline pain, and use shared decision-making [1,2,3,10,15].
Renal or hepatic impairment	Reduced clearance or altered exposure; higher vulnerability to rhabdomyolysis.	Dose adjust; avoid high-risk combinations; check CK/renal function when symptomatic [15,16,17,18,19,20,21].
Untreated hypothyroidism	Independent cause of myalgia and CK elevation; may amplify statin toxicity.	Check TSH when symptoms are unexplained, or CK is elevated; treat before rechallenge.
Polypharmacy: CYP3A4 inhibitors, cyclosporine, gemfibrozil, colchicine, selected antivirals/azoles/macrolides	Increased systemic statin exposure or additive myotoxicity.	Prefer lower-interaction statins; temporarily hold or reduce dose during short courses [22,23,24,25,26,27].
*SLCO1B1*/*ABCG2*/*CYP2C9* risk genotypes	Higher statin exposure, especially with simvastatin or rosuvastatin, depending on the gene–drug pair.	Use CPIC/DPWG guidance in selected patients; avoid overinterpreting genetics alone [38,39,40,41].
High-intensity statin or rapid dose escalation	Dose-dependent exposure and muscle complaints.	Use the lowest tolerated statin dose plus ezetimibe, bempedoic acid, or PCSK9-directed therapy if needed [45,46,47,48,49,50,51,52].
Medication fear, previous intolerance label, media exposure	Nocebo/drucebo amplification of real symptoms.	Normalize background muscle pain; explain blinded-trial evidence; use symptom diary or rechallenge [6,7,8,9,33,34].

## Data Availability

No new datasets were generated or analyzed during the current study. The evidence summarized in this narrative review was derived from publicly available published literature and guidance documents, all of which are cited in the reference list.
